# Quality of life of elderly ischaemic stroke patients one year after thrombolytic therapy. A comparison between patients with and without thrombolytic therapy

**DOI:** 10.1186/1471-2377-12-61

**Published:** 2012-07-26

**Authors:** Leonie de Weerd, Gert-Jan R Luijckx, Klaas H Groenier, Klaas van der Meer

**Affiliations:** 1 Department of General Practice, University of Groningen, Antonius Deusinglaan 1, 9713 AV, Groningen, The Netherlands; 2 Department of Neurology, University of Groningen, Hanzeplein 1, P.O. Box 30.001, 9700 RB, Groningen, The Netherlands

## Abstract

**Background:**

An observational study to examine whether thrombolytic therapy in stroke patients realizes better quality of life outcomes compared to patients without thrombolytic therapy one year after stroke. We also examined whether daily functioning, mental functioning and activities improved after thrombolytic treatment.

**Methods:**

A total of 88 stroke patients were interviewed at home one year post-stroke. Health-related quality of life (HRQOL) was assessed using the RAND-36, disability with the Barthel Index, depression and anxiety with the Hospital Anxiety and Depression Scale, and a questionnaire about patient way of life was completed. People aged under 60, moving to a nursing home or with a haemorrhage were excluded.

**Results:**

The thrombolysis group (TG) had more severe stroke (higher NIHSS) scores and were younger than the group without thrombolytic therapy (WTG). The primary outcome was HRQOL, which was high and nearly identical in both groups, however the TG had significantly better HRQOL for the ‘mental health’ and ‘vitality’ scales. Patients who stopped or reduced their hobbies because of stroke had a significantly worse HRQOL.

One year after stroke, more patients in the TG were totally or severely ADL dependent (12% TG and 0% WTG, p = 0.022). The level of dependence decreased in the TG (p = 0.042) and worsened in the WTG (p < 0.001) after one year. Being more dependent is related to diminishing daily occupations in both groups. In the TG the level of dependence had less impact on visiting family and friends and going on holiday. The prevalence of anxiety disorder and depression was low compared to other studies and there is no significant difference between the two groups.

**Conclusion:**

No major differences in the primary outcome (HRQOL) could be found between the two groups. In addition, no essential difference could be found in mental functioning and participation. We expected that patients undergoing thrombolytic therapy would have worse quality of life because of the greater initial severity of their stroke. Therefore, thrombolytic therapy seems to be of great importance in achieving better quality of life in ischemic stroke patients who respond to this therapy.

## Background

Acute ischaemic stroke is the second most common cause of death worldwide and a major cause of disability [1,2]. Each year, 19,000 men and 22,000 women in the Netherlands suffer a stroke [3]. The average age of patients suffering a stroke is seventy in men and seventy-five in women [4]. The elderly are particularly affected by strokes. Because of future aging it is important to pay greater attention to prevention and aftercare for stroke patients [5,6].

Currently, the interventions available to improve outcomes after ischaemic stroke are: admission to a stroke unit, the use of aspirin within 48 hours after stroke onset and treatment with intravenous tissue plasminogen activator (tPA) within 4.5 hours after stroke onset. Of these interventions, thrombolysis is the most effective and has dramatically changed attitudes towards and the management of stroke patients [7].

However, only a small percentage of ischaemic stroke patients receive tPA [8]. ‘The Netherlands stroke survey’ showed that 7% of all patients referred to a hospital with acute ischemic stroke ultimately received thrombolytic therapy [9]. Lack of patient familiarity with stroke symptoms and correct responses, late hospital arrival, the narrow therapeutic window and variance in the selection of patients for thrombolysis are among the factors that contribute to this under-treatment [7]. A feared complication of thrombolytic therapy is intracranial haemorrhaging. Symptomatic intracranial haemorrhage occurs in 1.7 to 8.0% of treated patients [7]. Despite this complication, thrombolytic therapy is still very effective and there is no reason to withhold this treatment from stroke patients, even elderly patients [10]. Stroke patients treated with tPA have after a year an almost twice as good a chance of a good functional outcome and are more likely to have minimal or no disability compared to those without such treatment [11].

A stroke affects patients’ lives in many different ways, not only physically but also through a range of emotional, psychological, cognitive and social consequences. The seriousness of post-stroke physical and mental impairments influences quality of life. As stroke mortality declines, more patients have to live with multiple handicaps and impairments. Therefore, improving the quality of life and paying greater attention to rehabilitation is increasingly important [6,12,13].

After discharge from hospital about 50% of patients return to their homes. Their general practitioners (GP) assist patients in coping with disabilities and psychological problems and manage secondary prevention [14]. Little is known about the long-term outcomes for Dutch patients after stroke, especially when compared to patients treated with thrombolysis. Most studies concentrate on functional outcomes in the acute or rehabilitation phase [15]. Accordingly, it is important for GPs to be aware of the primary care outcomes and what contributes to a good prognosis after a stroke [6,12]. In addition, it is important to know what the differences are between patients who received thrombolysis and patients who did not receive this kind of treatment.

As little is known about late physical and psychological consequences of ischemic stroke in primary care patients in the Netherlands, it is hard to determine whether patients are receiving quality aftercare and to determine whether thrombolysis treatment is of added value for a longer period after stroke.

The purpose of this study is to examine the quality of life one year after stroke of patients treated with thrombolysis compared to patients without thrombolytic therapy who returned to their homes immediately after discharge from hospital. We expected patients with thrombolytic therapy to experience a worse quality of life because of the greater initial severity of their stroke. Secondary outcomes include functioning, daily occupations, anxiety and depression.

## Methods

### Study design

The study included two groups of patients, namely all the ischemic stroke patients admitted to the University Medical Center Groningen, the Netherlands (UMCG) who were treated with thrombolysis between November 2007 and November 2008, and all the ischemic stroke patients admitted to a community hospital, Martini Hospital Groningen, the Netherlands, who were not treated with thrombolysis, between November 2006 and November 2007. We selected patients from two different hospitals because the UMCG acts as a comprehensive stroke centre to which patients from the region eligible for thrombolytic treatment are transported. The Martini Hospital acts as a community hospital where stroke patients ineligible for thrombolytic treatment are admitted.

The exclusion criteria for the study were being younger than 60 (because of this group’s profile in daily functioning and social life, and to create a more homogenous group), and referral to a nursing home, rehabilitation centre or another hospital department after being discharged from the hospital (because the GP stops being responsible for medical care under such circumstances). People already living in a retirement home before their stroke could enter the study. The study was approved by the MEC (medical ethical committee) of the UMCG and informed consent was obtained from all patients.

After informed consent, all patients were visited at home one year after stroke. Patients were interviewed in person by a trained medical practitioner and standardized questionnaires were completed. Clinical details – including stroke severity, comorbidity and vascular risk factors before stroke, medication and demographic information – were obtained from the medical records. Stroke severity was determined by the National Institute of Health Stroke Scale (NIHSS) [16].

### Measures at 12 months

The Barthel Index (BI) was used to assess disability in the patients. The BI measures the level of independence in ADL and yields a score ranging from 0 (functionally totally dependent) to 21 (functionally totally independent). In our questionnaire, patients were awarded three points for eating independently rather than two points in the original questionnaire. The sensitivity and reliability of the BI are high for stroke patients [17,18].

We used the RAND-36 to measure health-related quality of life (HRQOL). The RAND-36 consists of 36 questions and comprises 8 health scales (physical function (FF), role limitations physical (Rlf), social functioning (SF), role limitations emotional (Rle), bodily pain (BP), general health (GH), vitality (Vit) and mental health (MH)). The health scales range from 0 (poor HRQOL) to 100 (good HRQOL). The RAND-36 is a reliable and valid measure for determining HRQOL in stroke patients [19-21].

To identify the possible and probable presence of depression and anxiety disorders in our patients, the Hospital Anxiety and Depression Scale (HADS) was used. The HADS is a 14-item scale divided into depression and anxiety subscales. The possible scores for depression or anxiety range from 0 to 21. A score of 8 to 10 corresponds to possible anxiety disorder or depression and a score of 11 or higher indicates the probable presence of a mood disorder [22,23].

In addition, patients were asked about changes in their habits and daily occupations after their stroke, such as smoking, alcohol consumption, housekeeping, physical exercise, hobbies, reading, visiting family and friends, membership of clubs or associations and going on holiday.

### Statistical analysis

SPSS 15 for Windows (SPSS Inc., Chicago) was used for statistical analysis. Statistical significance was set at p < 0.05 (2-sided). For comparisons between groups, we used the following non-parametric tests: Mann–Whitney, Kruskall-Wallis and Analysis of Variance (ANOVA) using the Rank Transform method. The Rank Transform method consists of replacing the observations by their ranks in the combined sample and performing one of the standard analysis of variance procedures on these ranks [24]. The differences between groups were corrected for age and seriousness of stroke as a covariate. Because of the skewed distribution of the severity of stroke scale, the scores for this scale were converted to percentile ranks and then to normal curve equivalent (NCE) scores. Fisher’s exact test was used for categorical variables.

To describe the number of patients who changed their habits due to stroke, their responses were categorized into ‘more’, ‘less’, ‘as much as before’ or ‘quit’.

Despite the relatively large number of statistical tests applied, we decided not to correct for ‘multiple testing’ (for instance by means of the Bonferroni method). Instead, the p-values are simply presented as an indication of the strength of the evidence. Furthermore, since all patients admitted to the two hospitals during the study period were included, no formal power analysis was performed.

## Results

### Baseline characteristics

**Figure 1:**
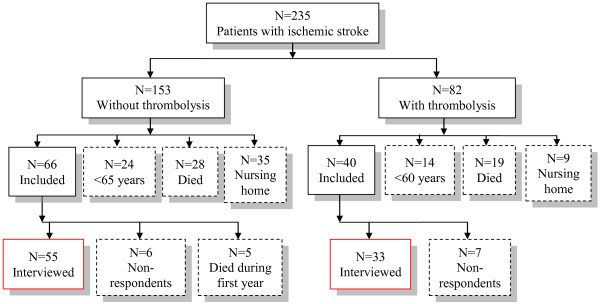
Flow chart of selection of patients.

In total, two hundred and thirty-five patients were diagnosed with ischemic stroke, of which eighty-two patients received thrombolytic treatment. Ultimately, eighty-eight interviews were conducted, with the reasons for exclusion shown in Figure 1. In the TG there were seven patients who could not participate because one patient was admitted to the intensive care unit at time of interview, one patient was living in Germany, two patients could not be contacted and three patients did not want to participate. In the WTG six patients could not participate, because one patient was rehabilitating from a hip fracture and the five other patients did not feel it necessary to participate because they were doing fine.

The detailed baseline characteristics of the study population are provided in Table 1. In the thrombolysis group there are significantly more men, the mean age is lower, the NIHSS is higher, more patients had strokes to the left hemisphere and more patients were living with a partner. In the following results we corrected for stroke severity and age.

**Table 1:** Baseline characteristics of the study population

**Variables**	**Thrombolysis (%)**	**Without (%)**	**p**
Patients included	33	55	
Gender^*^			**0.008**
- *Men*	24 (72.7)	32 (58.2)	
- *Women*	9 (27.3)	23 (41.8)	
Age, average (range)^#^	73.6 (61-85)	77.2 (65-91)	**0.025**
Living situation^*^			
- *Living alone*	7 (21.2)	30 (55.5)	**0.003**
- *Living with a partner*	26 (78.8)	25 (45.5)	
Brain hemisphere^*^			
- *Left side*	20 (39.4)	16 (29.1)	**0.007**
- *Right side*	13 (60.6)	34 (61.8)	
- *Unknown*		5 (9.1)	
NIHSS, average (range)^#^	9.97 (3-27)	2.6 (0-6)	**<0.001**
Length of hospital stay in days	7	7	1.000
Risk factors present before stroke/History^*^	1 (3.0)	6 (10.9%)	0.248
- *No risk factors*	23 (69.7)	27 (49.1)	0.076
- *Hypertension*	13 (39.4)	6 (10.9)	**0.003**
- *Hypercholesterolemia*	4 (12.1)	12 (21.8)	0.392
- *Diabetes*	9 (27.3)	16 (29.1)	1.000
- *Smoking*	9 (27.3)	14 (25.5)	1.000
- *Ischemic heart disease*	9 (27.3)	13 (23.6)	0.801
- *Stroke/TIA*	7 (21.2)	5 (9.1)	0.122
- *Atrial fibrillation*	1 (3.0)	2 (3.6)	1.000

* Fisher’s exact test, ^#^One-way ANOVA.

### Health-related quality of life (RAND-36)

The primary outcome of this study was health-related quality of life. In Table 2 a comparison is made between the TG and the WTG with respect to HRQOL. HRQOL is nearly identical in these two groups, except for the scales ‘Mental Health’ and ‘Vitality’. Patients in the thrombolysis group have a significantly better HRQOL for these two scales (p = 0.024, p = 0.024, resp.).

**Table 2:** Rand-36 comparison in the two study groups

**Rand-36**	**Thrombolysis**	**Without**	
	***Median (range), [95% CI]***	***Median (range), [95% CI]***	p^*^
*Physical functioning*	65 (0-100) [50-85]	62.5 (5-100) [55-80]	0.762
*Social functioning*	100 (0-100) [75-100]	100 (25-113) [75-100]	0.716
*Role limitations – physical*	50 (0-100) [25-75]	75 (0-100) [50-75]	0.346
*Role limitations – emotional*	100 (0-100) [100-100]	100 (0-100) [100-100]	0.467
*Mental Health*	88 (20-100) [80-96]	80 (16-100) [72-84]	**0.024**
*Vitality*	85 (5-100) [65-90]	65 (15-95) [60-70]	**0.024**
*Bodily Pain*	89.8 (0-100) [69.4-100]	89.8 (12-100) [69.4-100]	0.892
*General Health*	65 (0-100) [55-80]	65 (0-100) [55-75]	0.427

^*^Mann–Whitney test.

In Table 3 the interaction effects between the groups and the change in activities with respect to the RAND-36 are given, to explore to what extent differences between the two treatment modalities are mediated by these variables. The results were corrected for differences in age and stroke severity. There are two significant interaction effects between physical activity and the treatment groups (TG and WTG) for the health scale ‘Role limitations – emotional’ (p = 0.011) and between reading and the treatment groups for the health scale ‘Vitality’ (p = 0.033).

**Table 3:** RAND-36 interactions between the TG and WTG and activities

**HRQOL**	**Activities**	**p**^**#**^
**Physical functioning**	*Housekeeping*	0.374
	*Physical activity*	0.465
	*Hobbies*	0.327
	*Visiting*	0.615
	*Reading*	0.077
**Social functioning**	*Housekeeping*	0.145
	*Physical activity*	0.308
	*Hobbies*	0.264
	*Visiting*	0.077
	*Reading*	0.227
**Role limitations – physical**	*Housekeeping*	0.469
	*Physical activity*	0.868
	*Hobbies*	0.062
	*Visiting*	0.397
	*Reading*	0.657
**Role limitations – emotional**	*Housekeeping*	0.678
	*Physical activity*	**0.011**
	*Hobbies*	0.877
	*Visiting*	0.089
	*Reading*	**0.020**
**Mental health**	*Housekeeping*	0.076
	*Physical activity*	0.057
	*Hobbies*	0.638
	*Visiting*	0.583
	*Reading*	0.089
**Vitality**	*Housekeeping*	0.518
	*Physical activity*	0.104
	*Hobbies*	0.602
	*Visiting*	0.806
	*Reading*	**0.033**
**Bodily pain**	*Housekeeping*	0.498
	*Physical activity*	0.355
	*Hobbies*	0.164
	*Visiting*	0.898
	*Reading*	0.200
**General health**	*Housekeeping*	0.386
	*Physical activity*	0.221
	*Hobbies*	0.053
	*Visiting*	0.880
	*Reading*	0.560

p^#^ corrected for stroke severity and age.

^#^ Interaction rank transform ANOVA.

### Level of dependence according to the barthel index

At discharge from hospital, 70% of TG and 98% of the WTG are not dependent or slightly dependent (p = 0.001). One year after stroke this percentage is 88% (TG) and 96% (WTG) (p = 0.022). Immediately after discharge, 13% of the TG was severely or totally dependent; after one year this percentage was 12%. In the WTG none of the patients were totally or severely dependent at discharge or after one year.

When we compare the mean Barthel Index after one year, there is no significant difference between the TG (M = 18.03) and the WTG (M = 18.76) (p = 0.620). The mean Barthel Index in the TG after one year (M = 18.03) is significantly higher than at discharge from hospital (M = 15.97) (p = 0.042) and significant lower (M = 19.89 at discharge and M = 18.76 after one year) (p < 0.0005) in the WTG.

Being more dependent is related to diminishing daily occupations (significant for housekeeping in both groups (p < 0.05). An experienced decline in QOL is not related to being more dependent (Table 4).

**Table 4:** Functioning according to demographic factors, change in activities and aftercare

**Variables**		**Thrombolysis**			**Without**			
		***N***	***Median BI (range)***	p*	***N***	***Median BI (range)***	p*	***p***^***#***^
*Gender*^*2*^	Male	24	20 (4-20)	0.310	23	20 (12-20)	0.155	0.227
	Female	9	19 (9-20)		32	19 (13-20)		
*Social situation*^*2*^	Living alone	7	18 (18-20)	0.355	30	19.5 (13-20)	0.405	0.419
	Living with partner	26	20 (4-20)		23	20 (12-20)		
*Stroke location*^*2*^	Left side	13	20 (9-20)	0.561	34	20 (13-20)	0.851	0.096
	Right side	20	20 (4-20)		16	19.5 (12-20)		
	Unknown	0			5	20 (18-20)		
*Housekeeping*^*1*^	More	1	20 (20-20)	**0.005**	5	20 (18-20)	**<0.001**	0.612
	As much as before	21	20 (18-20)		25	20 (13-20)		
	Less	7	20 (8-20)		16	18 (15-20)		
	Quit	4	9 (4-18)		8	17 (12-19)		
*Physical activity*^*1*^	More	1	20 (20-20)	0.107	2	20 (20-20)	**0.003**	0.139
	As much as before	16	20 (18-20)		18	20 (16-20)		
	Less	15	20 (8-20)		30	20 (12-20)		
	Quit	1	4 (4-4)		4	14 (12-17)		
*Hobbies*^*1*^	More	0		**0.005**	1	20 (20-20)	0.662	0.866
	As much as before	17	20 (18-20)		34	20 (13-20)		
	Less	7	20 (18-20)		18	20 (12-20)		
	Quit	9	18 (4-20)		1	18 (18-18)		
*Visiting*^*1*^	More	1	20 (20-20)	0.058	0		**0.001**	**0.002**
	As much as before	25	20 (9-20)		37	20 (15-20)		
	Less	5	20 (8-20)		14	18.5 (13-20)		
	Quit	2	6.5 (4-9)		3	15 (12-17)		
*Vacation*	More	0		0.182	2	20 (20-20)	**0.007**	**0.001**
	As much as before	18	20 (9-20)		25	20 (13-20)		
	Less	9	20 (9-20)		12	20 (18-20)		
	Quit	6	18.5 (4-20)		15	17 (12-20)		
*Experienced quality of life*^*2*^	Increased	2	20 (20-20)	0.107	0		0.505	0.169
	The same	16	20 (18-20)		37	20 (13-20)		
	Diminished	15	19 (4-20)		17	19 (12-20)		

p^#^ corrected for stroke severity and age.

^*^ Kruskall-Wallis^1^ and Mann-Whitney^2^ test, ^#^ Interaction rank transform ANOVA.

Using the Rank Transform ANOVA, we investigated whether there were any interaction effects between the treatment modality (TG or WTG) and demographic factors and changes in activities with respect to the BI scores, to explore to what extent differences between the two treatment modalities are mediated by these variables. Moreover, all analyses were corrected for age and stroke severity. There are only two significant interaction effects, namely for visiting family and friends (p = 0.002) and vacation (p = 0.001). Changes in these two factors led to higher BI scores for the T patients compared to the WT patients (Table 4).

### Mood disorder (HADS)

In this study, 3% of the TG and 3.6% of the WTG had a history of depression.

One year after stroke, 9.1% of the TG and 5.3% of the WTG had probable depression. Additionally, depression is possible in 6.1% of the TG and 7% of the WTG. However, there is no significant difference in the prevalence of depression between the two groups (p = 0.055).

According to the HADS scores, one year after stroke, 6.1% of the TG and 5.3% of the WTG probably had an anxiety disorder. About 12.1% of the TG and 3.5% of the WTG possibly had an anxiety disorder. However, no statistically significant difference in prevalence between the two groups could be found (p = 0.634).

### Daily occupations

The percentages of patients who had to stop or diminish different activities are given in Table 5. There are no statistically significant differences between the two groups, except for hobbies. Significantly more patients in the TG had to stop their hobbies after stroke.

**Table 5:** Change in the frequency of daily occupations

**Daily Occupations**		**Thrombolysis (%)**	**No Thrombolysis (%)**	p*
*Physical exercise*	More	3.0	8.8	0.434
	The same	63.6	43.9	
	Less	21.2	28.1	
	Quit	12.1	14.0	
*Housekeeping*	More	3.0	3.5	0.546
	The same	48.5	31.6	
	Less	45.5	52.6	
	Quit	3.0	7.0	
*Hobbies*	More	0.0	1.8	**0.002**
	The same	51.5	59.6	
	Less	21.2	31.6	
	Quit	27.3	1.8	
*Visiting*	More	3.0	0.0	0.463
	The same	75.8	64.9	
	Less	15.2	24.6	
	Quit	6.1	5.3	
*Holidays*	More	0.0	3.5	0.544
	The same	54.5	43.9	
	Less	27.3	21.1	
	Quit	18.2	26.3	
*Reading*	More	3.0	3.5	0.398
	The same	69.7	77.2	
	Less	24.2	10.5	
	Quit	3.0	3.5	

* Fisher’s exact test.

## Discussion and conclusion

### Discussion

In this study we examined quality of life of ischaemic stroke patients one year after thrombolytic therapy compared to patients who had not undergone thrombolytic therapy.

Unfortunately, the two groups are not fully comparable. The severity of the stroke experienced by the patient group that underwent thrombolytic therapy was significantly greater than in the group without treatment. A possible explanation is that more severe stoke patients reach the hospital earlier and are eligible for thrombolysis [25,26]. The WTG was older and there were more women in this group, possibly because women may be less likely to reach hospital for thrombolysis treatment in time [27,28]. However, we corrected for the differences between the two groups. Risk factors present before stroke were approximately the same in both groups.

The primary outcome was health-related quality of life one year post-stroke. HRQOL in both groups was as high as in the Dutch elderly population [21] and is nearly identical in both groups, although patients in the thrombolysis group have a significantly better HRQOL for the ‘Mental health’ and ‘Vitality’ scales. Other studies report lower HRQOL after one year [29-31]. However, these studies included all patients with ischemic strokes, so they possibly studied more severely impaired stroke patients. It is known that higher ADL independence correlates to a worse HRQOL [29-32]. One possible explanation for the high scores on the RAND-36 is that a stroke has such an impact on life that patients one year post-stroke see quality of life in greater perspective. During their interviews, patients made comments such as: ‘it could have been worse’.

We examined whether there was a difference between the two groups in stopping or diminishing different activities. There was only a significant difference with respect to hobbies. This is possibly due to the fact that patients in the TG are younger and had more hobbies that they could practise before their strokes. Patients who stopped or reduced various activities have a significantly worse HRQOL. To increase HRQOL further, brief psychosocial intervention and antidepressant treatment could reduce post-stroke depression and improve functional outcomes [6,33]. Starting community-based rehabilitation programmes could also help by increasing the patients’ activity levels and give them greater satisfaction [34,35].

One year after stroke, more patients in the TG are ADL dependent. It is remarkable that one year after stroke, the average score on the BI in the TG has become higher than at discharge from hospital and lower in the WTG. This effect could be due to thrombolysis treatment [1,36]. Another explanation is that there are significantly more men in the TG. Some studies show that men are more likely than women to achieve functional independence [27,37].

Being more dependent is related to diminishing daily occupations in both groups [6,38]. It is important to pay attention to this in rehabilitation [6,39].

There were two significant interaction effects with respect to BI between the TG and WTG, namely for visiting family and friends and going on holiday. In the TG the level of dependence was of less influence on visiting family and friends and going on holiday. This could be because patients in the TG are younger. In the WTG there is a significant relationship between ADL independence and loss of social contacts. This has also been observed in other studies [6,40]. It is important to consider this in the rehabilitation of stroke patients because life satisfaction is significantly related to social activity and ADL independence [41,42].

After one year there is no significant difference in the prevalence of depression between the TG and the WTG. There is an indication of depression in about 12-15% for both groups. This is less than other studies indicate, namely 18-60% [43-45]. There also is no significant difference between the two groups with respect to anxiety disorder. About 9-18% have an anxiety disorder. This percentage is also less than was found in other studies, namely 25-50% [43]. It therefore seems that thrombolysis has no apparent effect on the prevalence of depression or anxiety disorder. It is nonetheless important to screen patients for depression or anxiety disorder because this significantly influences their quality of life [6,46]. By treating these diseases, HRQOL can be influenced positively.

In this study we used the HADS to measure depression and anxiety disorder, meaning that the percentages reported are only an indication. To diagnose these diseases, more extensive tests should be performed.

This study has several limitations. First, the study groups were relatively small because of the selection criteria. As a consequence, only large differences in the outcome variables resulted in statistically significant results. Another study also reported good health-related QOL for patients after thrombolysis [13]. Therefore, we expected minor differences in QOL after thrombolysis compared to those who did not receive this treatment. We may need to study a larger group of patients to detect a significant improvement in QOL after thrombolysis. Moreover, we only interviewed patients in the TG who went home immediately after discharge from hospital. They were probably good responders to thrombolytic therapy. We did not include those patients in the TG who were not discharged home and who were probably non-responders to thrombolytic therapy.

Secondly, the participants were distributed to two groups depending on which hospital they were admitted to, because the UMCG acts as a comprehensive stroke centre to which patients from the region eligible for thrombolytic treatment are transported. The Martini Hospital acts as a community hospital where stroke patients ineligible for thrombolytic treatment are admitted. Although the aftercare available to the two groups was the same, we could not completely rule out the confounding factor ‘hospital’. For stronger generalizations, the study group should be larger and patients from multiple hospitals with and without thrombolytic treatment should be studied.

One of this study’s strengths is the method of data acquisition. We visited patients at home to complete the questionnaires together. A lot of information was obtained through conversation, not only through the actual talking but also by being able to demonstrate.

Another strength is the fact that a single researcher visited all the patients and that we used standardized questionnaires. This avoided different interpretations of the results.

### Conclusion

To conclude, this study found no essential differences in health-related quality of life between patients with or without thrombolytic therapy. Independence increased in the patients with thrombolytic therapy. We expected that patients with thrombolytic therapy (with initial worse stroke severity) would have worse HRQOL, functioning/mental functioning and participation. Therefore, thrombolytic therapy appears to be of great value to achieving good quality of life in ischemic stroke patients. Perhaps quality of life can be improved further for both groups if aftercare is more specific. This needs further investigation in stroke patients.

## Competing interests

The authors declare that they have no competing interests.

## Authors’ contributions

LW, GJL and KM initiated the study. LW wrote the protocol. GJL and KM supervised data collection. LW, GJL, KG and KM wrote the manuscript. LW and KG performed statistical analysis. All the authors have read and reviewed the final manuscript.

## Pre-publication history

The pre-publication history for this paper can be accessed here:

http://www.biomedcentral.com/1471-2377/12/61/prepub
